# Drug resistance reversal: an overview of real world success stories

**DOI:** 10.1093/genetics/iyag253

**Published:** 2026-10-01

**Authors:** Pleuni S Pennings

**Affiliations:** ISEM, Université de Montpellier, CNRS, IRD, EPHE, Montpellier, Campus Triolet, Montpellier Cedex 05 34095, France

**Keywords:** antibiotic resistance, drug resistance, HIV, malaria, *E. coli*, *S. aureus*, MRSA, reversal evolution

## Abstract

This perspective provides an overview of what we know from real-world data on reversal of drug resistance evolution. An important question for those who study drug resistance is whether it is possible to reverse the evolution of resistance, for example by halting the use of specific drugs for a patient or even a community or country. Evidence for successful reversal of resistance levels is spread out in many different subfields of infectious disease. I use examples from *P. falciparum* malaria, several bacterial species including *E. coli*, *M. tuberculosis*, and *S. aureus*, and the viral pathogen HIV, to show that it is possible to reverse the evolution of resistance.

## What does reversing the evolution of resistance mean?

We use drugs to fight pathogens. But pathogens can evolve resistance, and when an allele or a gene that confers resistance to a certain drug has reached a considerable frequency within a host or in a population, the drug loses some, or all, of its usefulness.

It is important to try and prevent the emergence and spread of resistance genes and alleles. But once resistance to a certain drug is common in a pathogen population, it is also important to determine whether and how it is possible to reduce the fraction of resistant pathogens back to a lower level.

In this paper I focus on the fraction of a pathogen population that carries a resistance allele or gene and whether that fraction can be brought down by interventions (see Box 1). The resistance fraction can be viewed at the within-host level or at the population level. A reduction of the level of resistance can be considered a reversal of evolution of resistance.

Box 1. **The “level of resistance” can mean different things**

There are at least 2 important quantitative descriptors of drug resistance that may both be referred to as the “level of resistance”.

One meaning of the “level of resistance” is a measurement of how an individual pathogen strain with resistance responds to the drug as compared to how the pathogen without the resistance responds. This can be measured, for example, in a change in the minimum inhibitory concentration (MIC). The M184V mutation in HIV increases the MIC for the drug 3TC by 1000-fold (Tisdale et al. 1993), which is a very high level of resistance. Another possible measure of resistance is how it increases clearance times in curable diseases. Mutations in the *Kelch13* gene in *Plasmodium falciparum* were found to increase the parasite clearance times from around 48 to 84 h (Dondorp et al. 2009).

The second meaning of the “level of resistance”—and the one this paper is focused on—is the fraction of the pathogen population carrying a resistance allele or gene. We can study pathogen populations at 2 main scales: a pathogen population within a host or a pathogen population at the between-host level. For example, one can find that 15% of the viral particles in a blood sample from an HIV-infected person carry the K103N resistance mutation (Loubser et al. 2006). Or, one can determine that 15% of HIV samples collected from a cohort of patients carries the K103N resistance mutation (Machnowska et al. 2019). Typically, studies focus either on the within-host or the between host level and not both at the same time.

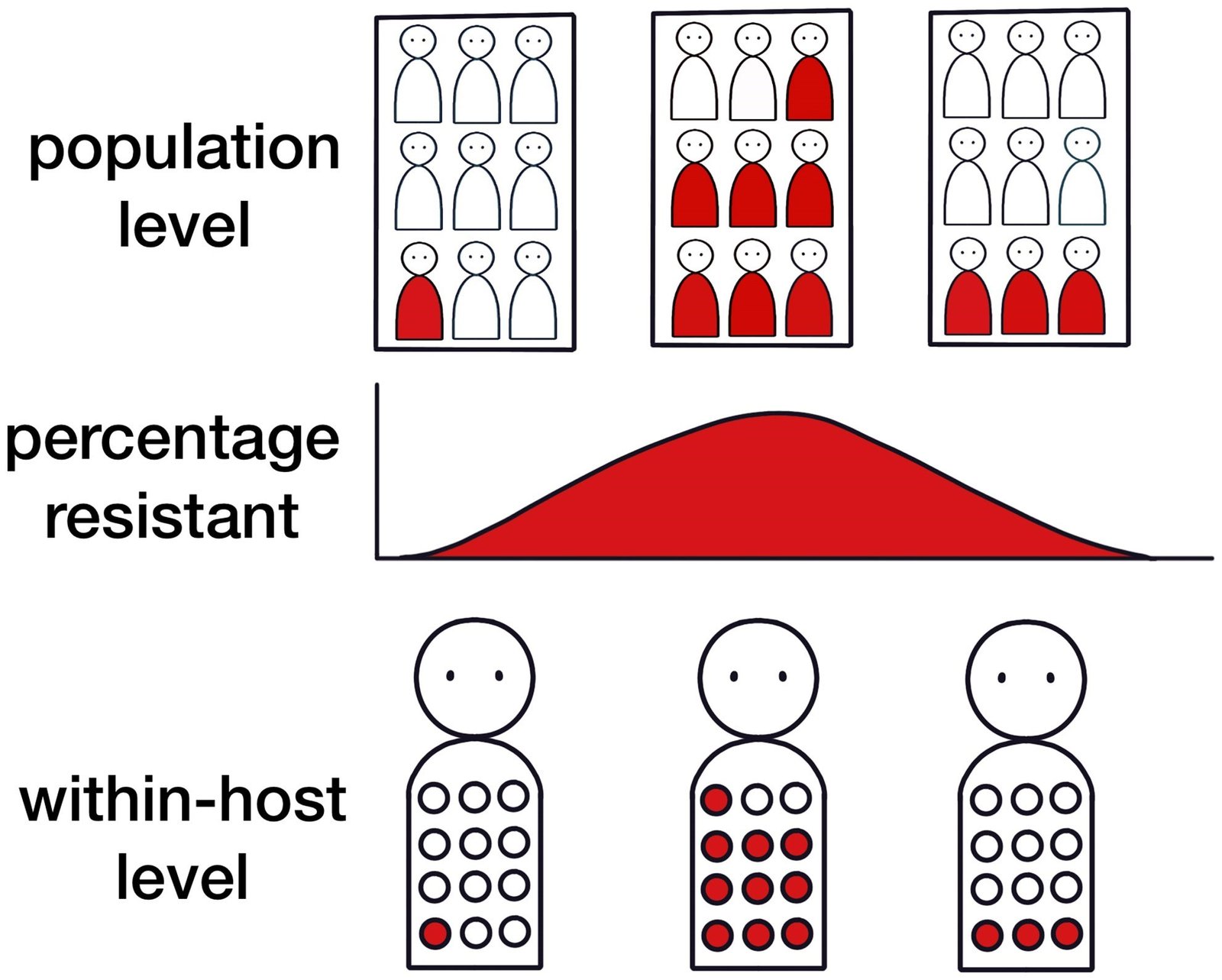
Box Image legend: The frequency, level or percentage resistance in a pathogen population can increase and decrease over time. In all panels, time goes from left to right, the frequency, level or percentage of resistance first goes up and then goes down again. Top panel: red indicates a host infected with a resistant pathogen, white indicates a host with a nonresistant pathogen. Uninfected hosts are not shown. In the middle panel the *y*-axis shows the level of resistance. The lower panel shows resistance within a host. Red indicates pathogen individuals (cells, viral particles) with resistance, whereas white indicates pathogen individuals without resistance.

Most interventions that are meant to lead to a decline of the fraction resistance are interventions that reduce or halt the use of the drug in question. Other strategies are infection control focused on resistant strains, and combination treatments (for HIV and TB) to prevent new resistance evolution, which have also shown success in reducing resistance levels.

An important underlying parameter is the fitness cost of a resistance allele or gene. Generally speaking, if resistance comes with a cost, we can expect that resistance levels will go down when the drug is used less or not at all. But if there is no cost, there is no reason to expect resistance levels to go down.

At the within-host level, the best examples of successful reductions in the level of resistance in human pathogens are known in HIV (Loubser et al. 2006; Lockman et al. 2007; Paredes et al. 2009; Jain et al. 2011b). At the population-level, there are convincing examples in the malaria parasite *Plasmodium falciparum* and in bacteria like *E. coli* and *S. aureus*. In *P. falciparum*, the reversal of chloroquine resistance from nearly 100% to 0% in large parts of southern Africa is especially impressive (Kublin et al. 2003; Laufer et al. 2010). In *E. coli*, the ban on colistin in China led to significant reductions in colistin resistance (Shen et al. 2020; Wang et al. 2020). Levels of methicillin-resistant *S. aureus* (MRSA) have gone down over the last 2 decades which is probably due to better infection control (Pham et al. 2024).

In this paper I provide examples of successful reductions in resistance in human pathogens to establish that reversal of resistance evolution is possible.

Note that this article is focused on success stories because I believe they have not gotten enough attention over the last decade. However, I do not mean to imply that interventions are guaranteed to lead to significant reductions in resistance. Failures happen too. However, a full analysis of such failures is complex and does not fit in this article for 2 reasons. First, there is likely a publication bias that needs to be taken into account. When an intervention is not successful, the results may not be published or even noticed. Second, there could be many reasons for a lack of success, including biological reasons (e.g. lack of cost of resistance) and nonbiological reasons (e.g. lack of compliance with a new policy).

## Malaria at the population level

We will start here with an example from *Plasmodium falciparum*, the most dangerous of the malaria parasites. *P. falciparum* is a eukaryotic parasite that can be found in many parts of the world. It is transmitted by mosquitoes and leads to a large number of deaths especially in Sub-Saharan Africa. For several decades in the 20th century, chloroquine was the main drug that was used to treat and prevent malaria. Starting in the 1950s, resistance to chloroquine evolved and spread, and it reached high frequencies in many countries, see Fig. 1a (Wernsdorfer 1991). In many areas the drug became useless. As a result, one by one countries decided to stop using the drug and in many countries the effect of banning chloroquine was that resistance levels decreased. The first country in Africa to ban chloroquine was Malawi and a clear reversal to susceptibility was observed in the 10 yr after the ban (Fig. 1b).

**Fig. 1.**
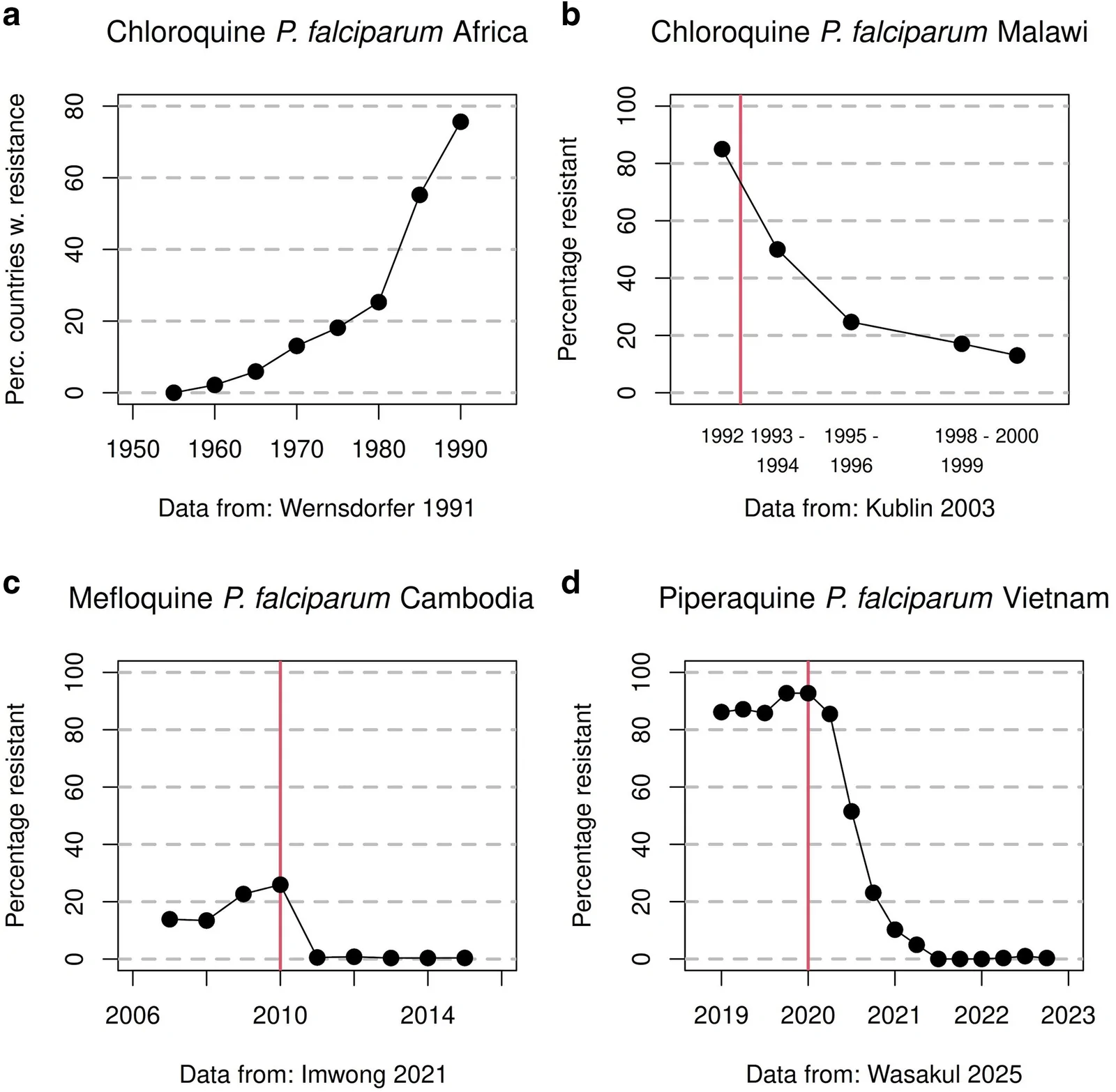
Resistance levels in *P. falciparum* malaria. a) The rapid spread of chloroquine resistance amongst countries with *P. falciparum* malaria. By the 1990s chloroquine resistance was present in most countries with malaria. Phenotypic data from Wernsdorfer (1991) captured with webplotdigitizer (Rohatgi 2010). b) The reversal of chloroquine resistance in Malawi, the first African country to ban chloroquine in 1993. Data on marker PfCRT K76T from Kublin et al. (2003) captured with webplotdigitizer (Rohatgi 2010). c) After Cambodia stopped using mefloquine as the first-line drug in 2010, resistance levels dropped very quickly. Data on the amplification of the PfMDR1 gene from Imwong et al (2021) captured with webplotdigitizer (Rohatgi 2010). d) After Vietnam stopped using piperaquine as the first-line drug in 2020, resistance levels dropped very quickly. Data on the amplification of the PfPlasmepsin2/3 from Wasakul et al (2025) captured with webplotdigitizer (Rohatgi 2010).

The peppered moth is one of the best known examples of an organism adapting to a changing environment. As the trees in England darkened because of pollution in the 19th century, so did the moths, and when the trees became lighter again in the second half of the 20th century, so too did the moths (Grant et al. 1996; Cook et al. 2012; van’t Hof et al. 2016). Chloroquine drug resistance in *P. falciparum* is like the dark color morph in peppered moths: when the drug was used extensively, resistance levels reached very high levels, but when the drug was removed, resistance disappeared again in many countries. An interesting parallel here is that just like the dark color morph in England, chloroquine resistance evolution in Africa was associated with a hard sweep as a single haplotype rose to high frequency (Wootton et al. 2002; van’t Hof et al. 2016). The return to majority wild type (susceptible strains in malaria and the light morph of the moths) came with the old genetic variation and is considered a soft sweep from standing genetic variation (Laufer et al. 2006; Mallet 2026).

Though the reversal of chloroquine resistance is best described for Malawi, resistance also decreased drastically in Zambia (Mulenga et al. 2021), Tanzania (Bwire et al. 2020), Kenya (Zhou et al. 2022), Mozambique (Galatas et al. 2017) and Zimbabwe (Mharakurwa et al. 2021). More moderate changes were seen in Ghana (Quashie et al. 2013), Nigeria (Amusan et al. 2024), India (Rana et al. 2022) and the Hainan province in China (Liu et al. 1995; Wang et al. 2005).

In South America, a return of susceptibility to chloroquine has been observed, but in that case it is not due to the return of the original wild type, but instead a new mutation recreated the susceptible phenotype (Pelleau et al. 2015), this mutation may also lead to resistance to another drug, so it is not clear what is creating the selection pressure for this mutation (absence of chloroquine or presence of the other drug, piperaquine), though either way the result is that chloroquine resistance is down.

In some parts of China, close to the Myanmar border (Hao et al. 2013; Bai et al. 2018), and in Vietnam, Laos and Cambodia (Wasakul et al. 2025) chloroquine resistance levels are still very high. It is not clear why resistance to chloroquine is still high in these places. It could be due to continued (informal) use of chloroquine, due to cross resistance, or due to a change of genotype-phenotype map (such as in South America). Finally, it could also be that there is no fitness cost of the resistance mutation or because there are no reversal mutants available.

After chloroquine was discontinued, several other antimalarial drugs were introduced and in some cases also discontinued because of resistance. In many countries, chloroquine was replaced by sulfadoxine/pyrimethamine and resistance to this drug combination evolved quickly (Kublin et al. 2003). Later the discontinuation of sulfadoxine/pyrimethamine as first-line therapy has not led to widespread reversal of resistance. This could be because this resistance possibly comes with no or little fitness cost or because the drug is still used for prevention which means that there could still be selection pressure for drug resistance (Bohissou et al. 2024; van Eijk et al. 2025).

Resistance also evolved to piperiquine and mefloquine which are used alone and in combination with other drugs (Kublin et al. 2003; Imwong et al. 2021; Wasakul et al. 2025). For both piperiquine and mefloquine, resistance appears to revert when the drugs are discontinued. In Cambodia, mefloquine was used before 2010 (as part of a combination therapy). When the drug was replaced by another drug, resistance levels went down quickly, see Fig. 1c (Imwong et al. 2021).

In Vietnam, resistance to piperaquine was very high (above 80%) but when the drug was replaced by another drug as first-line therapy, resistance dropped to near 0 in a year and a half, see Fig. 1d (Wasakul et al. 2025). These results suggest that resistance to mefloquine and piperaquine, which are both caused by the amplification of a gene, are very costly to the parasite.

## Bacteria at the population level

### Bans of drugs in farm animals can lead to dramatic reductions of resistance

The clearest examples of how reduced use of antibiotics can lead to reductions in resistance come from antibiotics use on farms. Sometimes antibiotics are banned in animals because its associated resistance can lead to treatment failures in humans. Such bans can lead to drastic reductions in resistance levels.

When it became clear in the 1990s that the use of the glycopeptide antibiotic avoparcin in farm animals was associated with glycopeptide resistant enterococci (GREs) in humans, the drug was banned in several European countries (Johnsen et al. 2009). After avoparcin was banned in Denmark in 1995, the level of resistance in *E. faecium* samples from chickens went down quickly; starting around 75%, going to 40% the next year and 20% the year after, ultimately reaching a new, but much lower, equilibrium between 0% and 5% a few years later, see Fig. 2a.

**Fig. 2.**
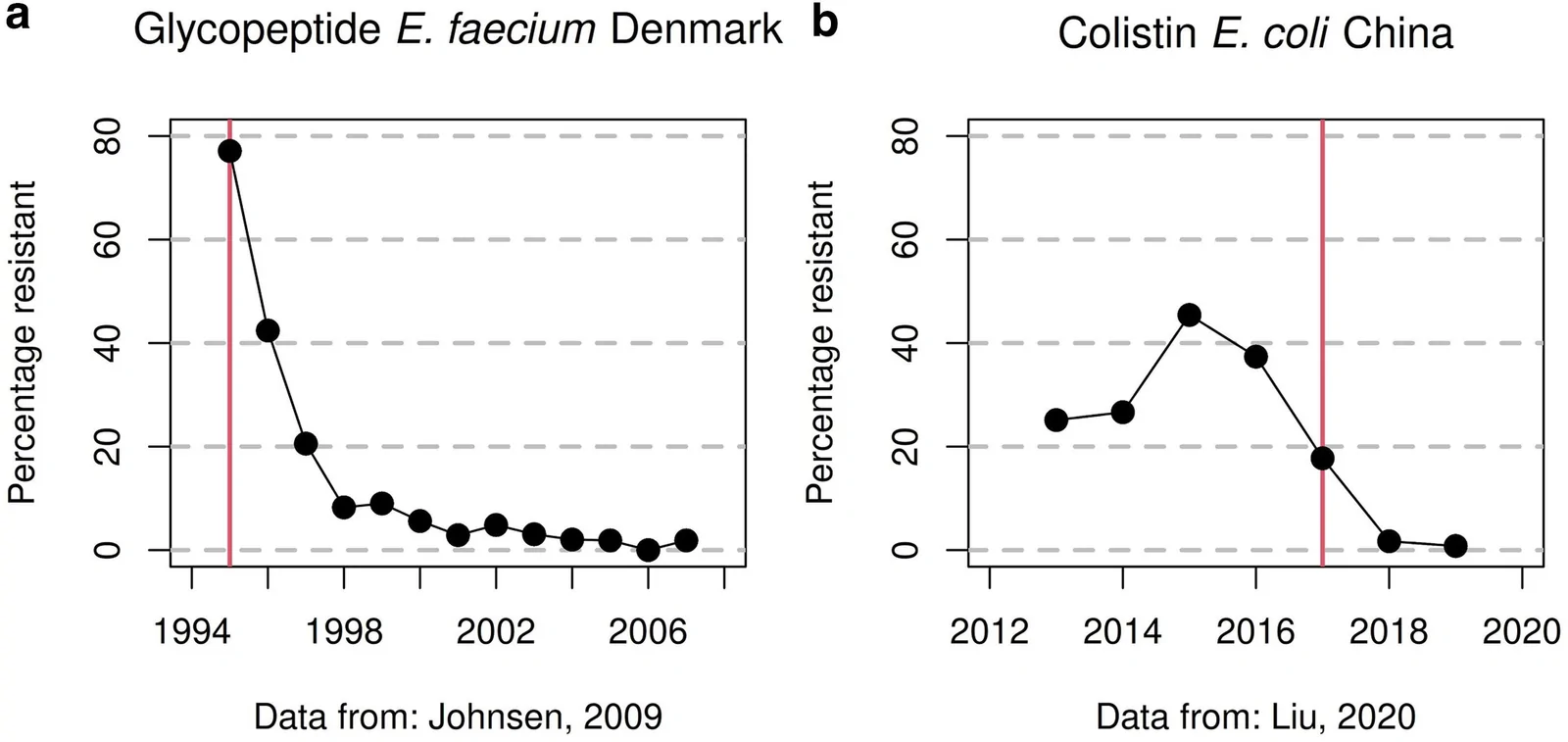
Resistance levels after bans in agricultural situations. a) Glycopeptide resistance in *E. faecium* in chickens in Denmark after a ban in 1995 (red line), data based on phenotypic tests from Johnsen et al. (2009) captured with webplotdigitizer (Rohatgi 2010). b) Colistin resistance in pigs in China after a ban in 2017 (red line). The ban went into effect in May of 2017. It is not clear why resistance already went down between 2015 and 2016, data from barplot of “colistin resistance” in Liu et al. (2020) captured with webplotdigitizer (Rohatgi 2010).

More recently, in the spring of 2017, the polymyxin drug colistin, which was commonly used for pigs, was banned in China. Liu and colleagues sampled *E. coli* from pigs before and after the ban (Liu et al. 2020). They found an ongoing reduction from nearly 50% in 2015 to below 5% in 2018, see Fig. 2b. Other, smaller scale studies show a decline of colistin resistance in Thailand, Japan and the UK after reductions in use (Randall et al. 2018; Usui et al. 2021; Khine et al. 2022).

### Large scale reduction of prescriptions: mixed effects

There are several well-known examples of cases where reductions in the use of a drug for humans did not lead to reductions in resistance. In the 1990s sulfonamide prescriptions were reduced by 98% in the UK from around 3 million in 1991 to 77,000 per year in 1999. Yet, resistance levels remained high in 1999 (Enne et al. 2001) and even in 2004 (Bean et al. 2005), see Fig. 3a. It is unclear why resistance to sulfonamides remained unchanged in *E. coli* but possible explanations are the absence of a cost of resistance, linkage with other resistance genes on a plasmid and continuing veterinary use of the drugs.

**Fig. 3.**
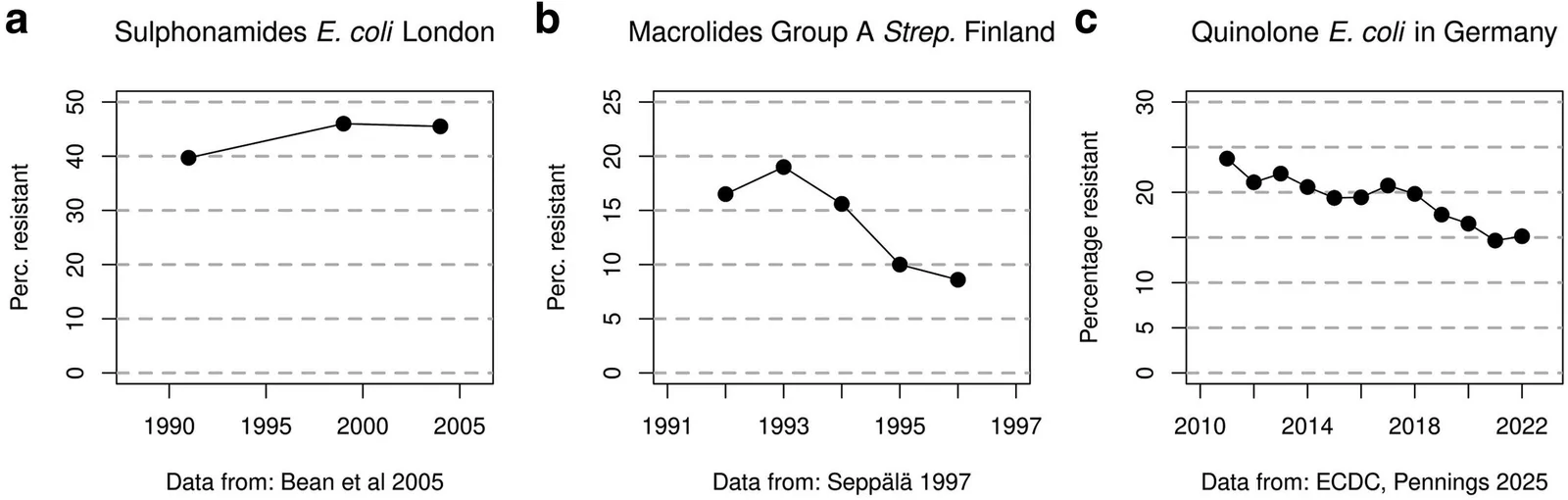
Resistance levels in bacteria in humans after reduced prescriptions. a) Sulfonamides prescriptions went down by 98% in the UK but resistance levels did not change in *E. coli.* It is one of the clearest examples of reduced prescriptions not affecting resistance levels, data on susceptibility tests from a table in Bean et al. (2005). b) In Finland, prescriptions to macrolides were reduced by ∼50% which led to a similar reduction of resistance levels amongst group A *streptococcal* isolates. Data on susceptibility tests from Seppälä et al. (1997). c) In 16 European countries quinolone prescriptions went down and in 15 of these 16 resistance levels went down in *E. coli*. Here shown are the data from susceptibility tests in Germany as described in Pennings (2025).

Another example of a large-scale reduction of prescriptions which did not lead to a reduction of resistance comes from an intervention study in Sweden. In this study there was one county that drastically reduced the use of the drug trimethoprim while a neighboring county did not (Sundqvist et al. 2010). In the intervention county, use of trimethoprim was reduced by around 85% for 2 yr, but the effect was minor: resistance had slightly decreased in the intervention county while it had slightly increased in the control county. Similarly, sustained and significant reduction in use of trimethoprim in England from 2015 till 2023 also only lead to a very slow reduction in resistance (Kettlewell et al. 2024).

Unlike these 2 cases where resistance levels did not change much, there are also examples where a reduction of use led to reduced levels of resistance. In 2001 in the wake of the 9/11 attacks and anthrax attacks in the USA, Israel restricted the use of quinolone drugs to preserve these drugs for possible anthrax attacks. Gottesman and colleagues recorded a reduction of the use of quinolones of 50%, and a decrease of quinolone resistance in *E. coli* from 12 to 9% (Gottesman et al. 2009). However, both the reduction in use and the reduction in resistance were short lived.

In Finland in the 1990s, there was a successful effort to restrict the use of macrolide drugs. Their use was reduced by around 50%, which was associated with a similar reduction of resistance among group A *streptococcal* isolates to the macrolide drug erythromycin. The biggest reduction in drug use happened from 1991 to 1992 (from 2.4 to 1.4 defined daily doses per 1000 inhabitants), which was sustained after. Resistance levels peaked in 1993 and declined afterwards, from 19% in 1993 to 8.6% in 1996 (Seppälä et al. 1997), see Fig. 3b.

I have studied the relationship between quinolone prescriptions and quinolone resistance in *E. coli* in Europe. I found that there is a consistent effect of the change of usage on the change of resistance. In 15 countries that reduced the number of prescriptions from 2011 to 2021, resistance levels went down whereas in 3 countries that increased the number of prescriptions, resistance went up. Only one country, Sweden, showed an unexpected effect: usage went down moderately and resistance went up somewhat (Pennings 2025), see Fig. 3c for the reduction in resistance in Germany.

### Prescription reductions in hospitals

Many studies have been done in individual hospitals and in groups of hospitals, to determine if reducing prescriptions will lead to lower levels of resistance in the hospital.

Schuts and colleagues did a meta-analysis on the effect of reducing drug use in hospitals (Schuts et al. 2021). They found evidence that reducing fluoroquinolone and piperacilline-tazobactam prescriptions can decrease resistance in nonfermenters, such as *P. aeruginosa* and *Acinetobacter* spp (Lee et al. 2018; Schuts et al. 2021). On the other hand, they found no evidence for an effect of reducing use of carbapenems or third-generation cephalosporins in Enterobacterales, nonfermenters, or Gram-positive bacteria (Schuts et al. 2021). However, this is a situation where the absence of evidence should not be taken as evidence of absence. There were relatively few studies in these categories and some had very little power. For example, some studies aimed to study both whether drug use changed and whether resistance levels went down, but when they determined that drug use did not change, it is no surprise that resistance levels did not go down either (Mach et al. 2007).

A newer study, which was not included in the Schuts review, showed an effect of reduced fluoroquinolone usage on resistance in both *P. aeruginosa* and *E. coli* (Swingler et al. 2022), see Fig. 4a. Another large new study focused on the US Veterans Affairs Healthcare System from 2007 until 2022. They found that fluoroquinolone prescriptions went down by more than 80% and resistance to fluoroquinolones went down in most of the pathogens surveyed (Pham et al. 2024), see Fig. 4b for the effect in *S. aureus*.

**Fig. 4.**
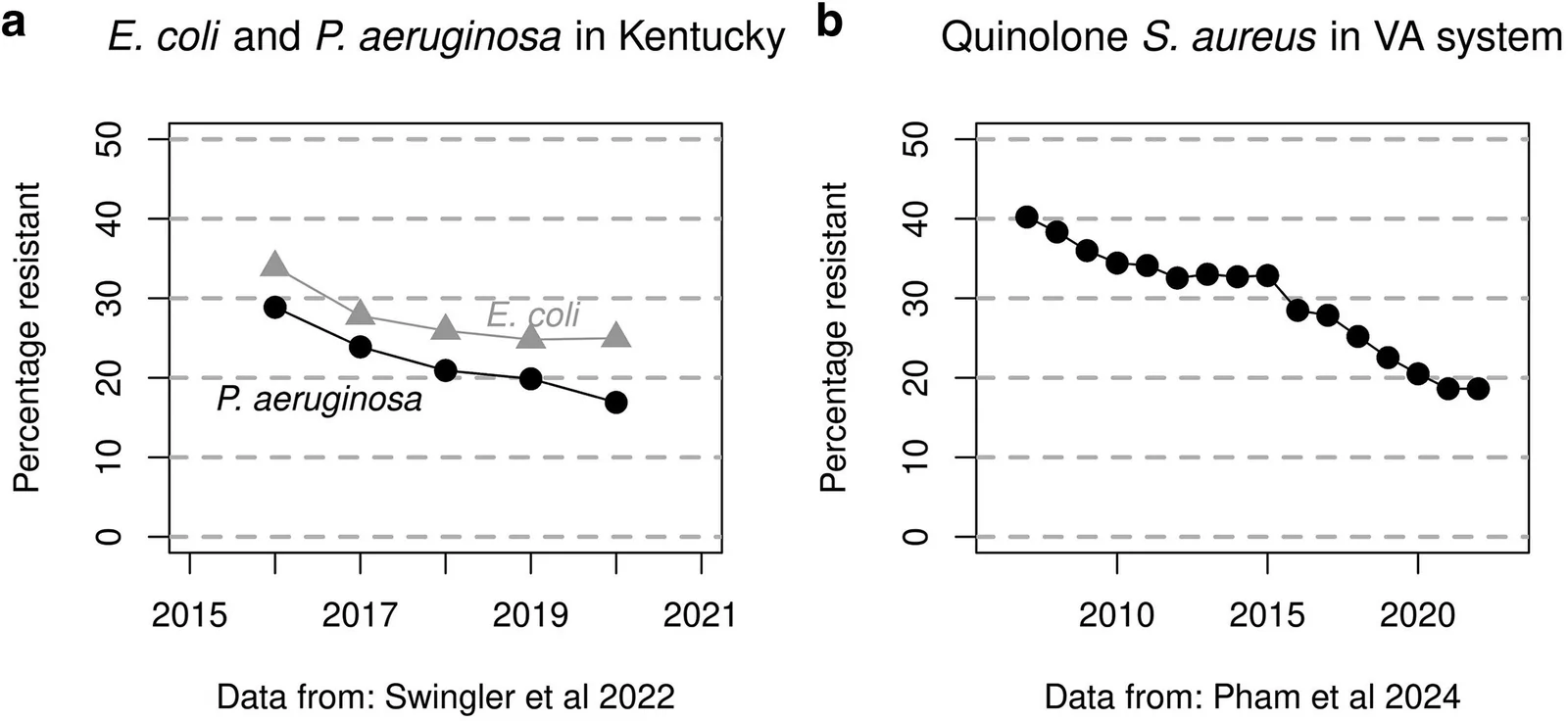
Resistance levels in bacteria in hospitals. a) Fluoroquinolone resistance in *E. coli* (gray triangles) and *P. aeruginosa* (black circles) in a hospital in Kentucky, USA. Data on susceptibility tests from Swingler et al. (2022) captured with webplotdigitizer (Rohatgi 2010). b) Fluoroquinolone resistance in *S. aureus* in the Veterans Affairs Hospital system in the US. Fluoroquinolone prescriptions went down and so did resistance to fluoroquinolones in most pathogens that were studied in Pham et al. (2024). Data on susceptibility tests captured with webplotdigitizer (Rohatgi 2010).

### Better use of drugs and infection control can also lead to reduced resistance

While reducing the use of drugs may be the most obvious and best-documented way to reduce levels of resistance, there are other interventions that have been shown to work. And in some cases, it turns out that using *more* drugs is the right thing to do to reduce the level of drug resistance and the number of infected people at the same time.

In the early 1990s, multidrug resistant tuberculosis (MDR TB) was a big problem in New York City with nearly 20% of the TB cases being MDR TB (Frieden et al. 1995). To cure people from active TB, a combination of drugs needs to be taken for several months. It is important that TB-infected people adhere to their treatment regimen because treatment interruptions are known to lead to drug resistance evolution in TB (Pablos-Méndez et al. 1997; Hirpa et al. 2013).

During the 1990s, New York City increased the budget for TB control and hired more people to improve the TB treatment infrastructure. One method to make sure that patients take their drugs every day is to enroll them into a directly-observed-therapy program (DOT) where a case worker visits the patient every day and the patient takes the drugs in the presence of the case worker. The number of people in a directly observed therapy program in New York increased from <50 in 1983 to more than 1200 in 1994 (Frieden et al. 1995). In addition to the DOT program, infection control was improved in hospitals and at Riker's Island (the jail for all 5 New York boroughs), and infections were reduced by having fewer people stay in large shelters. These efforts reduced the number of new cases of TB in general, but had an even bigger effect on TB cases resistant to one or more drugs, so that the fraction of cases that were resistant to any or multiple drugs went down (Frieden et al. 1995; Munsiff et al. 2006), see Fig. 5a.

**Fig. 5.**
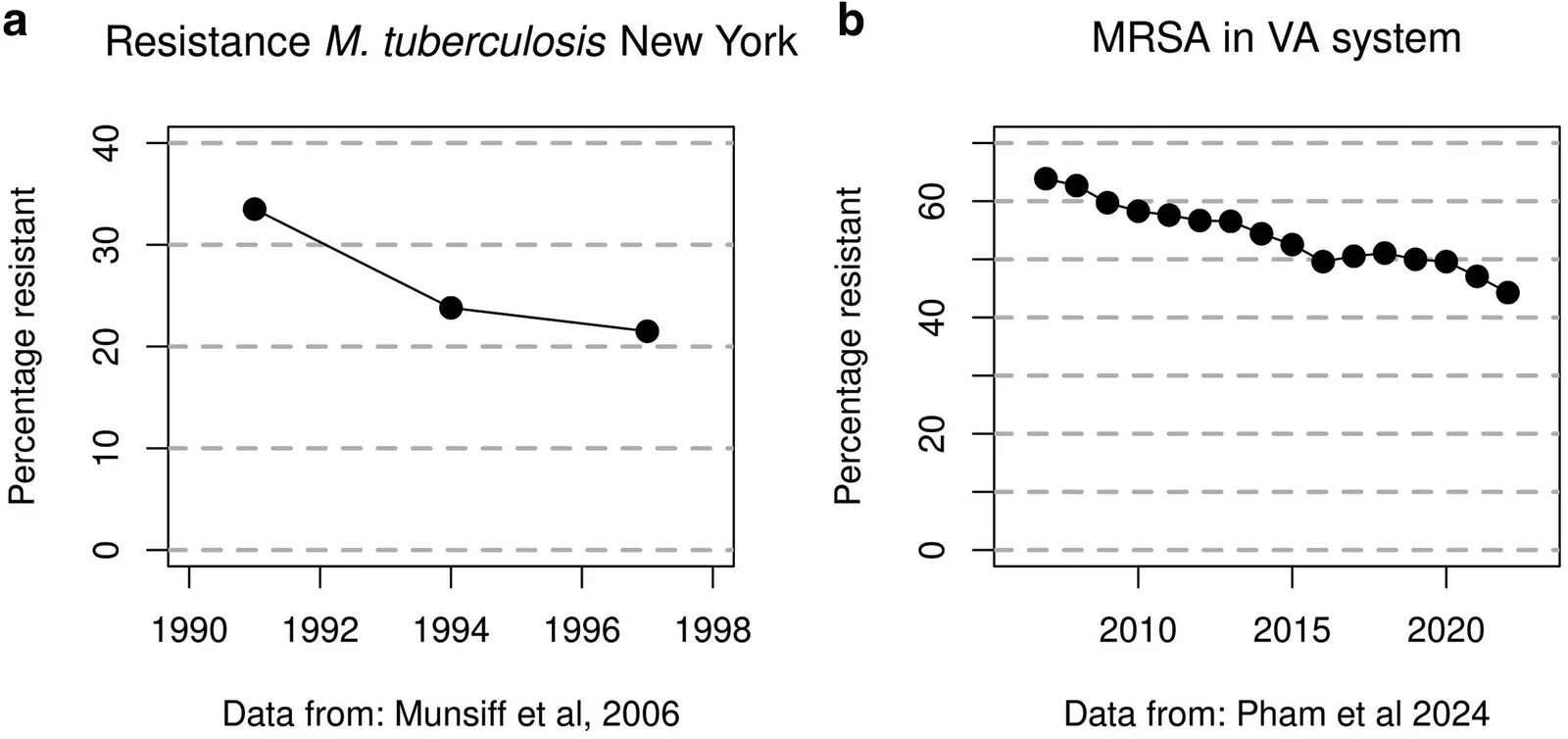
Resistance levels in tuberculosis and *S. aureus*. a) Increased access to DOT (directly observed therapy) and infection control in shelters, hospitals and jails led to a reduction in resistance to any drug amongst TB cases in New York City in the 1990s. Data on susceptibility tests from Munsiff et al 2006. b) The percentage methicillin resistance amongst hospital acquired *S. aureus* infections in the VA system in the USA went down from 2007 until 2022 due to an “MRSA bundle” which included improved surveillance, hand washing and other changes in the hospitals, data on susceptibility tests from Pham et al. (2024) captured with webplotdigitizer (Rohatgi 2010).

From a population genetic perspective there are several possible reasons for the reduction of resistant cases beyond the reduction of nonresistant cases in New York. One is that the infection control efforts were more likely to impact resistant cases (e.g. when resistant cases were concentrated in shelters, hospitals or Riker's Island). It is also known that DOT programs reduce the evolution of new resistant strains and transmission of resistance (Moonan et al. 2011).

Other studies have looked at improved treatment and isolation practices in hospitals. In the Veterans Affairs Healthcare system in the US improved surveillance, isolation practices, hand hygiene and hospital culture led to a reduction in the proportion of methicillin resistance in *S. aureus* from 57.7% to 44.6% from 2007 to 2019 (Jain et al. 2011a; Pham et al. 2024). Figure 5b shows the percentage methicillin resistance amongst hospital acquired *S. aureus* infections in the VA system from 2007 till 2022.

A review paper by Baur et al. shows that in many studies infection control can reduce the number of resistant bacteria infections in a hospital (Baur et al. 2017). However, for most of these studies it is not clear if the fraction of strains that carry resistance changes. So while some of the studies show impressive effects, we cannot make any conclusions about evolution in these cases.

## Reduced NRTI resistance in HIV at the population level

Amongst the different classes of HIV drugs, NRTI drugs are the oldest. The first of its kind, AZT, was approved in the US in 1987. In the late 1980s and early 1990s several NRTI drugs became available and people infected with HIV were treated with 1, 2 or even 3 NRTI drugs. However, within-host evolution of resistance happened quickly in almost all cases. This meant not only that treatment usually failed to improve patients’ health long term, it also had the secondary effect of the transmission of these resistant strains to others, leading to significant levels of resistance amongst newly infected people. The situation improved in the mid-1990s when 2 other drug classes became available (PI drugs and NNRTI drugs). From here on it was common to combine NRTI drugs with either a PI or an NNRTI drug to create triple-drug therapies also known as Highly Active Antiretroviral Therapy (HAART). One of the main results of using combinations of drugs was that resistance evolution became much less common (Feder et al. 2016; Rocheleau et al. 2018).

For NRTI drugs, the improved treatments not only led to less within-host evolution, but also to a reduction of transmitted resistance in countries that switched from NRTI-only treatments to triple-drug treatments around 1996. Figure 6 shows the trend for transmitted NRTI resistance in Germany. The reason for the reduction of transmitted NRTI resistance is likely that resistant strains lead to somewhat lower viral loads and thus have lower transmission potential (Paredes et al. 2009). In addition resistance can revert to wildtype within the host (see next paragraph) which also contributes to resistant strains not being transmitted as often as wild type stains (Jain et al. 2011b).

**Fig. 6.**
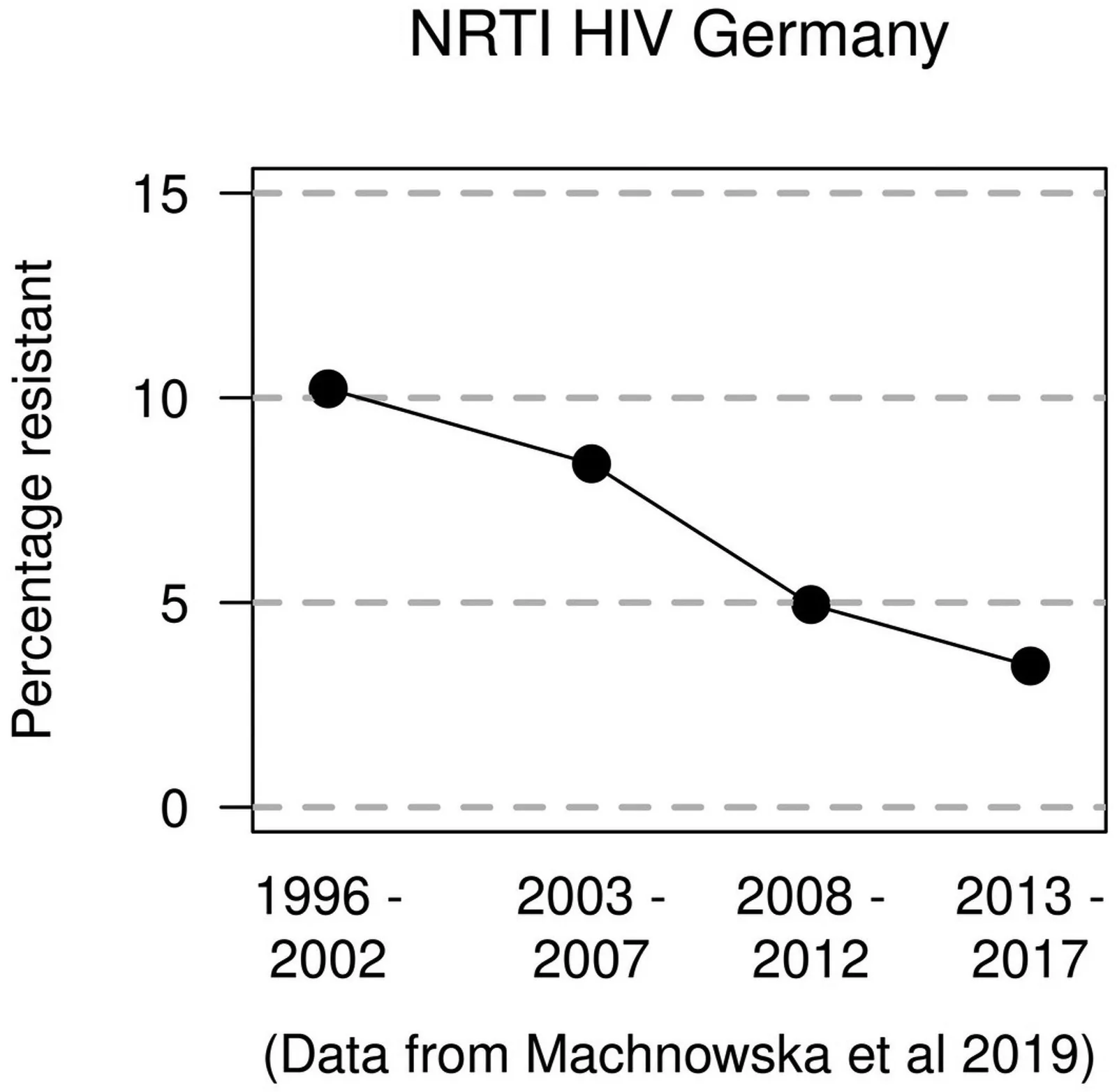
Reduction of NRTI resistance after introduction of triple-drug therapy in 1996, data based on genotypes and resistance predictions according to the Stanford HIVdb algorithm from Machnowska et al. (2019) captured with webplotdigitizer (Rohatgi 2010).

Since the mid-2010s, NNRTI and PI drugs have been replaced by INSTI drugs (integrase inhibitors) in many countries (Bangalee et al. 2022). It is likely that in these places levels of transmitted resistance to NNRTI and PI drugs will go down over time.

## Within-host reversal evolution

### HIV drug resistance reverts within host when treatment is halted

There is good evidence that resistance in HIV can be reversed within hosts. In the 2000s, a single dose of the NNRTI drug nevirapine was regularly used to prevent mother-to-child transmission of HIV. An unwanted side effect of this treatment was that within-host resistance levels would often go up quickly. This is likely because nevirapine resistance mutations are available as standing genetic variation prior to treatment starting, and because nevirapine has a long half-life so that the resistance mutation is positively selected for several weeks. Population-genetic studies showed that nevirapine resistance mutations like K103N revert in about 6 to 12 mo (Loubser et al. 2006) because of a mild cost of the resistance mutations, see Fig. 7a for an example.

**Fig. 7.**
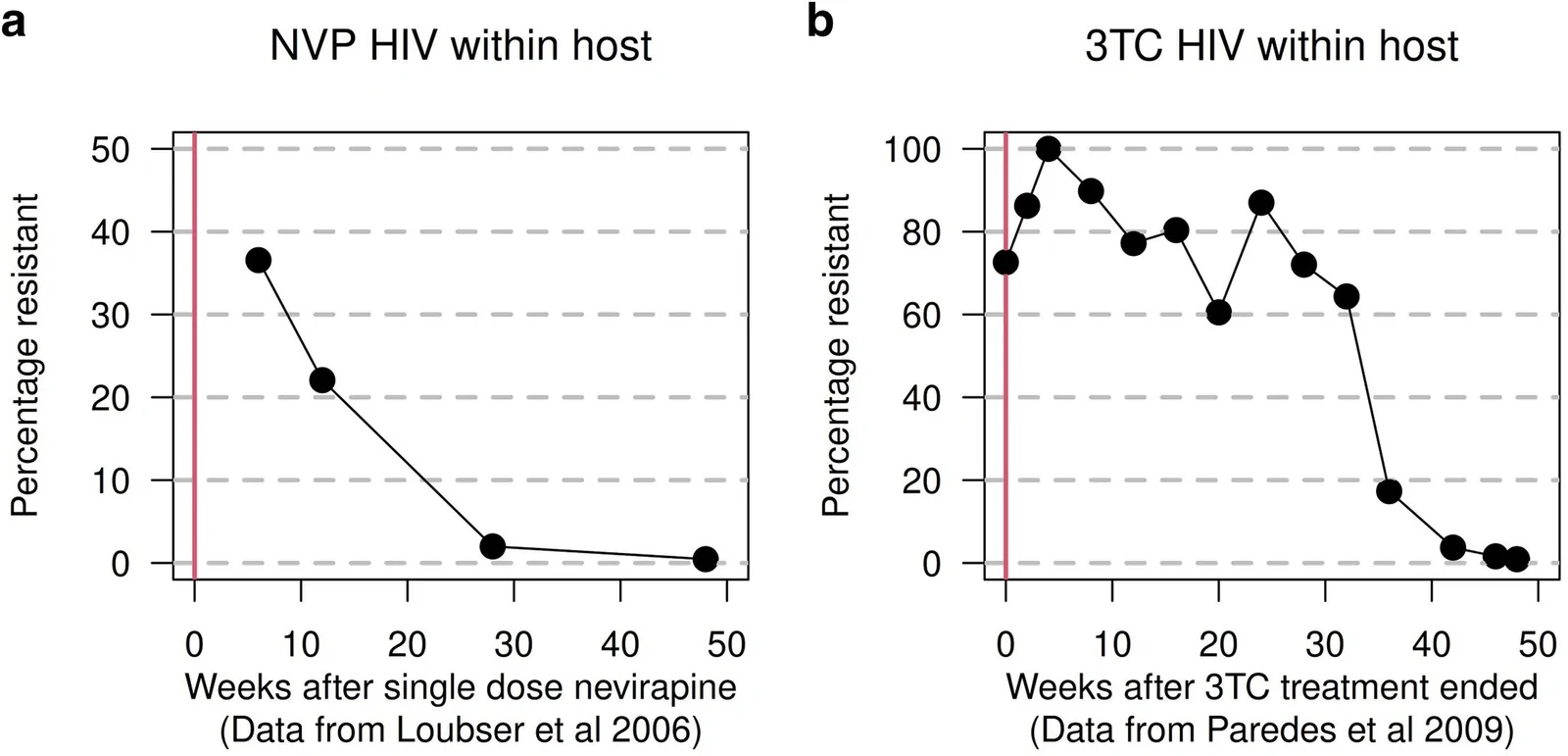
Resistance levels in HIV. a) Decay of nevirapine resistance (K103N mutation in the RT gene) within a host after a single dose of nevirapine at week 0 (vertical line). Data from Loubser et al. (2006) captured with webplotdigitizer (Rohatgi 2010). b) Decay of 3TC resistance (M184V mutation in the RT gene) after 3TC treatment ended at week 0 (red line). Treatment continued with other drugs. Data from Paredes et al (2009) captured with webplotdigitizer (Rohatgi 2010).

Clinical studies align with the population-genetic results and show that when NNRTI-based triple-drug therapy was started within 6 mo after a single dose of nevirapine such treatment was likely to fail due to resistance. On the other hand, if treatment was started later they did not fail more often than expected (Lockman et al. 2007).

Single-dose nevirapine is exceptional because, as the name implies, it is taken as a single dose (though sometimes 2 doses are taken). Most HIV drugs, however, are taken daily for months or years. If resistance evolves during continued treatment, the treatment should be switched to a second-line set of drugs. In those cases, does resistance revert? It turns out that it does, though not always immediately. Data from several studies suggest that it takes a variable number of weeks before the “right” reversal mutation or archived wild type virus appears. Then, once that is the case, resistance levels go down to near 0 in a predictable manner (Deeks et al. 2001; Paredes et al. 2009). It is not clear why reversal mutants are not available as standing genetic variation in these cases. See Fig. 7b for an example from Paredes et al. (2009).

Finally, there is the situation where someone gets infected with a resistant strain (transmitted resistance). In such cases, the wild type needs to be recreated by mutation before resistance levels can go down. There is good evidence that this happens, though it can take around 6 mo (median) for M184V and longer for other mutations, as shown in an elegant paper by Jain et al. (2011b).

### Little evidence and few studies on within-host reversal evolution in bacteria and malaria

Data on within-host evolution of resistance in bacteria and malaria parasites in humans are much less available than data on within-host resistance evolution in HIV. Studies on within-host reversal of resistance are especially hard to find. The influential review by Andersson and Hughes about reverting resistance evolution refers to 2 studies where resistance evolved in within-host bacterial populations and reversal did not happen even after a year or longer of no treatment (Andersson and Hughes 2010). A newer study by Yaffe and colleagues studied the evolution of quinolone resistance in gut bacteria in 60 volunteers who took a short course of the quinolone drug ciprofloxacin. In most participants, Yaffe et al. observe no reversal after 70 d, though there are a few exceptions, the most extreme one is shown in Fig. 8 (Yaffe et al. 2025).

**Fig. 8.**
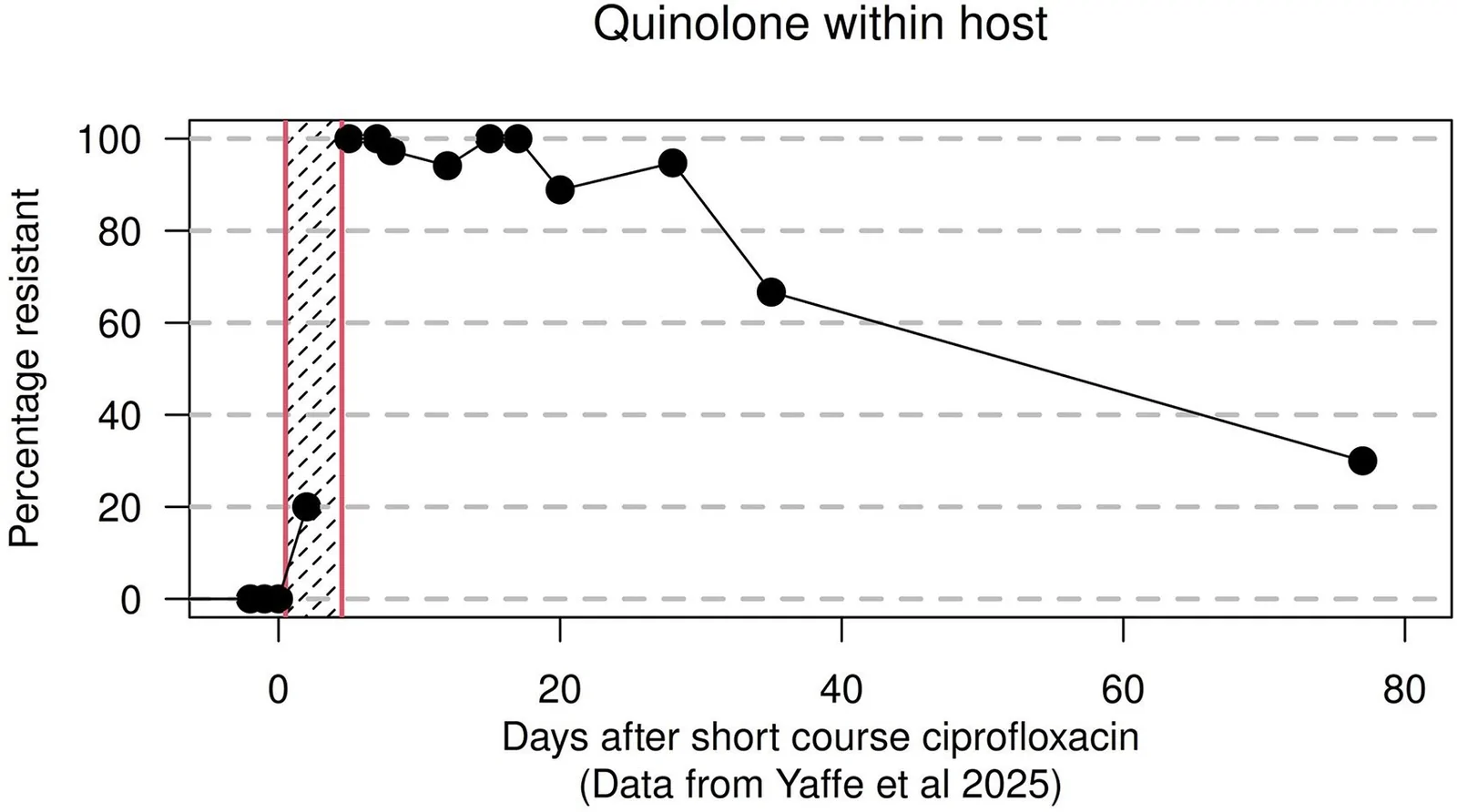
Reversal of quinolone resistance encoded by a mutation in the *gyrase A* gene in gut bacteria in volunteers who took 5 d of ciprofloxacin (indicated by the shaded area). Out of 61 detected sweeps involving the *gyrase A* gene in the study, this one showed the fastest reversion. Most showed no reversion at all. Data from Eitan Yaffe personal communication and Yaffe et al. (2025).

In the malaria parasite there is even less published data on within-host evolution of resistance in human hosts. One exception is a paper by Uhleman and colleagues that shows that copy numbers of *pfmdr1*, which are associated with resistance to mefloquine, tend to go up in patients treated with mefloquine. On the other hand, in patients with high pretreatment copy number who are treated with another drug (not mefloquine) the copy number tends to go down and thus resistance reverts in those patients (Uhlemann et al. 2007).

## Discussion

In this paper I have listed examples of the reversal of the evolution of drug resistance in human pathogens. Examples come from *P. falciparum* malaria, various bacteria including *M. tuberculosis*, and the viral pathogen HIV. There is evidence for reversal of resistance at the between-host (population) level and also at the within-host level. I have focused on these examples to show that reversal of resistance is possible. This paper is not meant to give a comprehensive overview of what happens when treatment policies are changed. In particular, I have only listed a few examples where resistance levels did not go down despite attempts, more such examples exist and could maybe be listed in a separate paper.

The presented examples of reversals make clear that reversal of resistance happens at different time scales. Even within the same system, time scales vary. For example, at the population level, chloroquine resistance in *P. falciparum* malaria took 10 or more years to significantly revert (Fig. 1b) while piperaquine resistance reverted in a year and a half (Fig. 1d). This is likely due to piperaquine resistance being more costly for the parasite than chloroquine resistance, though alternative explanations should be considered in future studies.

For the bacterial examples, it is not surprising that resistance at the population level reverts more quickly after a ban (e.g. colistin resistance Fig. 2b) than after a gradual reduction in the use of a drug (e.g. quinolone resistance Fig. 3c). For the within-host HIV examples, reversal takes between several weeks and a year and depends on the mutation (Deeks et al. 2001; Lockman et al. 2007; Paredes et al. 2009; Jain et al. 2011b). These differences in time scales could be due to many things including different generation times, treatments, geographic scales and fitness costs.

While this paper has focused on successful reversals of drug resistance evolution, it is clear from the literature that reversals do not always happen when we expect them. Even a near complete ban is no guarantee for reversal (for example, sulfonamide resistance in Fig. 3a). In cases where a reduction of the use of a drug is expected to lead to a reduction of resistance, there could be many reasons why this does not happen. First off, it could be that there is no fitness cost incurred by the resistance allele or gene. Second, it could be that there was originally a cost but over time the additional mutations have reduced or eliminated that cost (compensatory evolution). Third, it could be that selection *for* resistance continues because the drug is still used informally, still used in farm animals, or because the resistance allele or gene also confers resistance to another drug. Fourth, it could be that resistance is under selection in nearby areas such as neighboring countries, cities, hospitals, and migration is stronger than selection. And finally, it could be that there is no genetic variation, or that the resistance allele or gene is linked to other genes under selection. The issue of linkage hampering reversal of resistance is particularly relevant for bacteria where different resistance genes and virulence genes can co-occur on plasmids.

Conversely, when resistance levels consistently go down after the reduction of use of a drug, it likely means that first, there is a fitness cost, which second, is not eliminated by compensatory evolution, that third, the reduction in drug use is real, fourth, that migration is not stronger than selection and fifth, that there is genetic variation not hampered by linkage. It should be noted though that if reduced use of one drug is directly accompanied by the increase of use of another drug, it could be that selection against the resistant strains is due to negative cross resistance (also referred to as collateral sensitivity or double bind). Huijben et al. present an example of such a case in malaria (Huijben et al. 2020). For quinolone resistance, it is likely that there is a simple fitness cost because a reduction in levels has been seen after reduced prescriptions in several bacterial species (*S. aureus*, *E. coli*, and *P. aeruginosa*). Similarly, for most resistance mutations in HIV a fitness cost is likely the cause for reversals within the host.

Most of the examples in this paper are reductions of resistance levels after reduced use of drugs. However, there are also examples of reduced resistance due to improved adherence (*M. tuberculosis* in Fig. 5a), improved surveillance and hand washing (MRSA in Fig. 5b) and the introduction of triple-drug therapies (HIV in Fig. 6).

Given that it is clear that resistance levels *can* go down, research should focus on improving our understanding of the conditions under which resistance goes down as compared to the conditions where it doesn’t. Here I propose 3 comparisons that should be studied:

1. Chloroquine resistance went down in many countries after chloroquine use was halted, but in some countries, like Vietnam, resistance to chloroquine is still high. It would be of interest to study how these countries are different in terms of treatment policies and informal markets for drugs (Frosch et al. 2011) or how the parasites in these countries differ in terms of transmission dynamics, or genetically (Pelleau et al. 2015). It would also be of interest to compare chloroquine resistance with resistance to sulfadoxine/pyrimethamine (SP), which appears to not revert even when the drugs are no longer used (Bohissou et al. 2024; van Eijk et al. 2025).
2. Quinolone resistance has gone down in many places in several different bacterial species (Schuts et al. 2021; Pham et al. 2024; Pennings 2025). However, at the within-host level, it looks like it is very stable (Yaffe et al. 2025). It would be of interest to study this apparent contradiction.
3. There are 2 well studied cases where drug use was reduced but resistance did not go down (sulfonamides in London (Bean et al. 2005) and trimethoprim in Sweden and England (Sundqvist et al. 2010; Kettlewell et al. 2024)), these should be compared with the cases where resistance did go down (colistin (Liu et al. 2020), quinolones (Pennings 2025)).

How else will we make progress? I believe that genomics, including long-read sequencing and phylogenetic methods will help us better understand the evolutionary dynamics of resistant strains, including their cost and their origins (Whittles et al. 2017; Helekal et al. 2023; Tracy et al. 2024; Pennings 2025). In addition, continued surveillance of pathogens and systematic data collection on the use of drugs will help us understand patterns of resistance. I see a bright future for the field of drug resistance evolution and have good hope that science-backed interventions will continue to help keep resistance in check.
